# Electrical and Dielectric Properties of ZnO-BaO-V_2_O_5_ Glasses

**DOI:** 10.3390/ma19163456

**Published:** 2026-08-14

**Authors:** Tina Tasheva, Ondrej Bošák, Marian Kubliha

**Affiliations:** 1 Department of Silicate Technology, University of Chemical Technology and Metallurgy, 8 Kliment Ohridski blvd, 1797 Sofia, Bulgaria; 2 Faculty of Materials Science and Technology, Slovak University of Technology, Bottova 25, 917 24 Trnava, Slovakia; ondrej.bosak@stuba.sk (O.B.); marian.kubliha@stuba.sk (M.K.)

**Keywords:** vanadate glasses, ZnO, dielectric relaxation, electrical conductivity, Raman spectroscopy, impedance spectroscopy, glass structure

## Abstract

The relationship between composition, structure, and electrical properties is essential for understanding charge transport in vanadate glasses. In this study, the influence of ZnO concentration and thermal treatment on the structure, electrical conductivity, and dielectric relaxation of xZnO–(35 − x)BaO–65V_2_O_5_ glasses (x = 0–20 mol%) was systematically investigated. Two series of glasses, as-quenched and annealed, were prepared by the melt-quenching technique and characterized by temperature-dependent direct current (DC) conductivity, broadband dielectric spectroscopy, and Raman spectroscopy. The DC conductivity exhibited Arrhenius behavior with two thermally activated conduction regions. Annealing reduced the activation energy at higher temperatures, indicating structural relaxation and stabilization of the glass network. Dielectric spectroscopy revealed two distinct relaxation mechanisms over the frequency range of 0.1 Hz–100 kHz, whereas thermal treatment had only a minor influence on the dielectric relaxation behavior. Raman spectra showed that ZnO progressively modifies the vanadate network through the formation of V–O–Zn linkages while preserving the characteristic terminal V=O bonds. Annealing enhanced the short-range structural ordering without altering the fundamental glass structure. Among the investigated compositions, the glass containing 7 mol% ZnO exhibited distinct structural and electrical characteristics, suggesting that this composition corresponds to a transition in the structural organization of the glass network. The combined structural and electrical analyses demonstrate a clear correlation between ZnO-induced structural modifications and the charge transport properties of ZnO–BaO–V_2_O_5_ glasses.

## 1. Introduction

Oxide glasses have attracted significant scientific and technological interest because their structure can be modified over a wide compositional range, enabling the development of materials with tunable optical, electrical, magnetic, and dielectric properties. These characteristics make them promising candidates for applications in photonics, electronics, and energy-related devices [1,2].

Among transition-metal oxide glasses, vanadium-containing systems are of particular interest because vanadium can adopt multiple oxidation states (V^2+^–V^5+^) owing to its partially filled 3d electronic shell [3,4,5]. V_2_O_5_, in which vanadium exists predominantly as V^5+^, is the most thermodynamically stable vanadium oxide and is therefore widely used as a precursor for the preparation of vanadate glasses. Depending on the glass composition and melting conditions, vanadium ions may coexist in different oxidation states and coordination environments, mainly as VO_4_ tetrahedra and VO_5_ polyhedra, thereby strongly affecting the glass structure and its electrical, dielectric, and optical properties [3,4]. In glasses with relatively low optical basicity, partial reduction of V^5+^ to V^4+^ results in the coexistence of both valence states within the glass network. The coexistence of V^4+^ and V^5+^ ions gives rise to thermally activated electronic conduction via small-polaron hopping between neighboring vanadium ions, which governs the electrical conductivity and dielectric response of vanadate glasses [3,4,5,6,7]. Owing to its variable coordination and oxidation state, V_2_O_5_ is generally regarded as a conditional glass former that can function either as a network former or as a network modifier depending on the glass composition and local structural environment.

The incorporation of modifier oxides such as ZnO and BaO into vanadate glass networks provides an effective approach for tailoring both the glass structure and its functional properties [8,9,10,11,12]. Zinc oxide is particularly attractive because it may exhibit a dual structural role depending on its concentration, acting either as a network modifier or participating in the glass network through the formation of V–O–Zn linkages [12,13,14]. These structural modifications influence the distribution of vanadium species, defect concentration, charge-carrier mobility, and dielectric relaxation processes. Consequently, ZnO-containing vanadate glasses have attracted considerable interest for potential applications in electronic and energy-storage devices.

Although the structural and optical characteristics of ZnO-containing vanadate glasses have been investigated previously, the combined influence of ZnO substitution and thermal history on their DC conductivity, impedance response, and dielectric relaxation remains insufficiently understood. In particular, a systematic comparison of as-quenched and annealed xZnO–(35 − x)BaO–65V_2_O_5_ glasses over the composition range has not been reported. The present work therefore aims to establish correlations between composition, thermal history, short-range structural organization, charge transport, and dielectric relaxation using complementary electrical and Raman-spectroscopic methods.

## 2. Materials and Methods

Two series of xZnO–(35 − x)BaO–65V_2_O_5_ glasses (x = 0, 1, 3, 5, 7, 10, 15, and 20 mol%) were prepared to evaluate the influence of thermal history. The initial raw materials ZnO (99%), BaCO_3_(Alfa Aesar), and V_2_O_5_ (Alfa Aesar (Thermo scientific), Thermo Fisher, Kandel, Germany 99.2%) were homogenized in an agate mortar, and the batches with a size of 30 g were melted at 750 °C for 15 min in a porcelain crucible in an electric furnace and then melt-quenched by pressing between two plates to form disks. The first series comprised as-quenched samples, whereas the second series consisted of melt-quenched glasses subjected to a subsequent annealing treatment at 250 °C.

DC conductivity measurements were performed on the prepared samples twice in a row in a protective atmosphere of nitrogen gas. For the determination of the DC conductivity, electric currents were measured at a constant voltage of 1 V using Novocontrol Concept 90 (Novocontrol Technologies GmbH & Co. KG, Montabaur, Germany) temperature range from 20 °C up to 210–250 °C depending on glass stability. The current was determined by a picoammeter, Keithley 6517B (Tektronix, Beaverton, OR, USA). Temperature dependences of the DC conductivity were measured at increasing temperature, with a heating rate of 5 °C/min.

AC measurements (from 20 °C up to 250 °C) were done using LCR Hi-tester Hioki 3522-50 (Hioki, Ueda, Japan) at a frequency range 0.1 Hz–100 kHz in the Novocontrol NOVOTHERM system using a standard sample cell BDS 1200 (Novocontrol Technologies GmbH & Co. KG, Montabaur, Germany). Measurements were done in steps of 5 °C, after 20 min tempering at chosen temperatures. For the evaluation of the bulk DC conductivity, the frequency dependence of the AC conductivity was used. To avoid the influence of electrode polarization effects and to better understand electrical relaxation, impedance and modulus spectroscopy concepts were used [15].

The prepared glass samples in the form of plates with a thickness of ~1 mm and an area of up to 150 mm^2^ were ground and polished to remove any unwanted artifacts on the active surfaces of the samples and to ensure their plane parallelism and flatness. No other material was added to the contact surface of the samples.

The experimental uncertainty of electrical measurements (both AC and DC) was estimated from repeated measurements of the same specimens to be below 7% of the measured value. Repeated measurements of the dependence on selected glasses confirmed the stability of the values and repeatability of the measurements. The uncertainty of the measured temperature during electrical and dielectric characterization was below 1 °C.

The phase state of the samples was examined by X-ray diffraction using a PANalytical Empyrean diffractometer (PANalytical, Almelo, The Netherlands) in θ–θ geometry. The instrument was operated at 40 kV and 30 mA with Cu Kα radiation (Kα_1_ = 1.540598 Å and Kα_2_ = 1.544426 Å). The diffraction data were collected in continuous mode between 5.00 and 95.02° 2θ, with a nominal angular increment of 0.0525°. A fixed 0.5° divergence slit was used.

DSC curves were made at 10 °C/min using a STA PT 1600 TG-DTA/DSC LINSEIS Messgerate GmbH calorimeter (LINSEIS Messgeräte GmbH, Selb, Germany). The glass transition temperature Tg and crystallization temperature Tx were estimated from the DSC curves.

The Raman spectra were performed by Raman Renishaw inVia microscope (Renishaw, Glocestershire, UK) with Leica DM2700M (Leica Microsystems CMS GmbH, Wetzlar, Germany). Analysis conditions: laser: 532 nm, integration time: 10 s, laser power: 5 mW, 5 accumulations.

## 3. Results and Discussion

### 3.1. Powder X-Ray Diffraction

**Figure 1:**
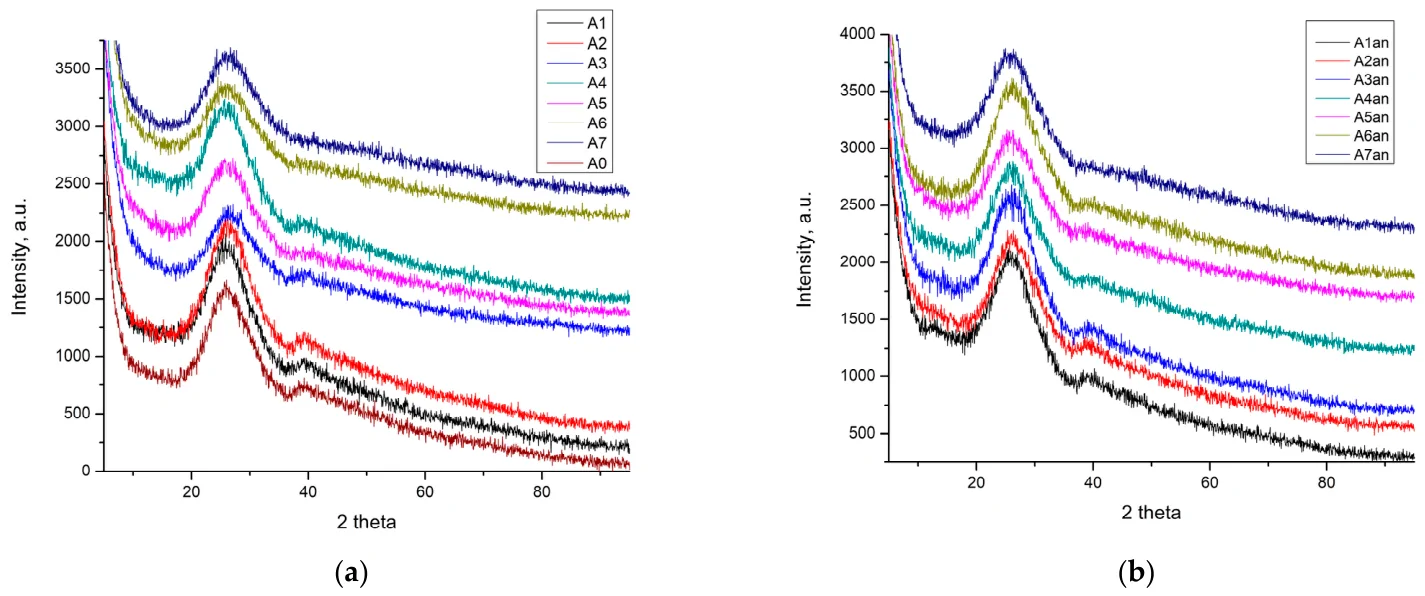
XRD patterns of the (**a**) as-quenched and (**b**) annealed samples.

The XRD patterns of the samples are presented in Figure 1. The profiles are characterized by a broad scattering band, while discrete Bragg reflections are not observed within the examined angular range. This diffraction response indicates the absence of long-range periodic ordering and confirms that the samples are amorphous. Possible differences in the position and shape of the broad band can be related to changes in the short- and intermediate-range arrangement of the glass network. Since such variations cannot be interpreted unambiguously by XRD alone, their structural origin is further evaluated using the spectroscopic results.

### 3.2. DSC Measurements

DSC measurements of the as-quenched glasses revealed glass-transition temperatures between 262 and 303 °C and onset temperatures of the first crystallization event between 315 and 372 °C (Table 1). The resulting thermal-stability parameter, $\Delta T=T_{x1}-T_{g}$, ranged from 50 to 73 °C. The maximum temperatures used for the electrical measurements (210–250 °C, depending on composition) were therefore below both the glass-transition and crystallization-onset temperatures. The reported DSC parameters refer only to the as-quenched samples [13].

### 3.3. DC Conductivity

**Figure 2:**
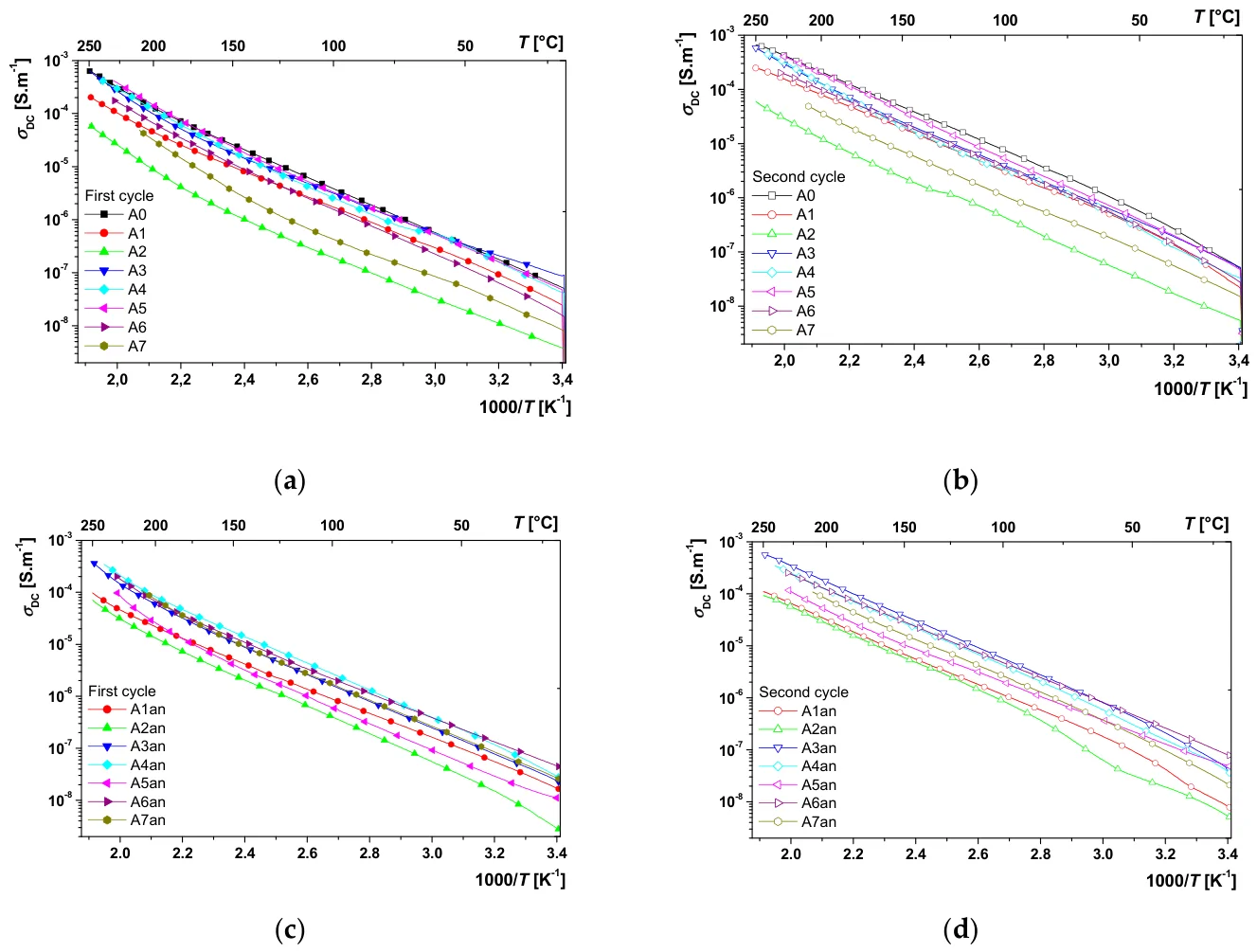
Temperature dependencies of the DC conductivity of the ZnO–BaO–V_2_O_5_ glass system: (**a**) the first heating measurement cycle, (**b**) the second heating measurement cycle, (**c**) the first heating measurement cycle of annealed glasses (250 °C), (**d**) the second heating measurement cycle of annealed glasses (250 °C).

Temperature dependences of the DC conductivity, σ_DC_, of the glasses are presented in Figure 2 (the first and second heating cycles). The measured dependences at the first heating cycle (Figure 2a) show the significant influence of the change in chemical composition. The conductivity values vary within 1.5 orders of magnitude depending on the ZnO concentration. Table 2 compares the measured values of direct electrical conductivity, σ_DC(100 °C)_, at 100 °C.

The measured temperature dependences of the DC conductivity of the glasses with two temperature intervals in Figure 2 show Arrhenius behavior, according to

*σ*_DC_ = *σ*_o_ exp(−*E*_σ_/*kT*) (1)

where σ_o_ is the pre-exponential factor, Eσ is the conduction activation energy, *k* is the Boltzmann constant, and *T* is thermodynamic temperature. For both temperature ranges (up to 120 °C and above 150 °C), the activation energy and pre-exponential factor values were determined using the least squares method. The temperature range of both intervals was determined based on the correspondence of the shape of the measured dependencies with the shape of the dependency according to relation (1) and controlled by the value of the correlation factor. As can be seen from Table 2, the activation energy value of the DC conductivity increases in the higher temperature range. The pre-exponential factor values also show changes in the higher temperature range.

In Figure 2b, the measured dependences of the DC conductivity of the glasses during the repeated measurement (the second heating cycle) are shown. When comparing the values during the first and repeated measurement (Figure 2a,b), there are noticeable differences. These are observable not only in the values of DC conductivity, but also in the values of the corresponding activation energies and pre-exponential factors (Table 2).

In the high-temperature region (above 150 °C), the chemical composition has a significant impact on the activation energy values. Generally, a decrease in activation energy is observed when comparing the first and second measurements of the glasses. At the same time, a decrease in activation energy is observed when comparing as-quenched and annealed glasses. An example of this effect is the glass with the composition 3ZnO–32BaO–65V_2_O_5_ (sample A2), for which an activation energy of 0.82 eV was observed during the first heating in the high-temperature region and 0.60 eV during repeated heating. For a glass of the same composition annealed during preparation (sample A2an), activation energy values of 0.62 eV in the first measurement and 0.54 eV in the repeated measurement were obtained. In this temperature range, the pre-exponential factor changes significantly, particularly when comparing the first and repeated measurements of glasses prepared without annealing.

In the temperature range up to 120 °C, minimal changes in the activation energy values are observed when comparing the first and repeated measurements, as well as annealed and as-quenched glasses. Starting from the composition 7ZnO–28BaO–65V_2_O_5_, a more significant increase in the activation energy is observed during repeated measurements for both annealed and as-quenched glasses (from 0.52 to 0.56 eV). For the 10ZnO–25BaO–65V_2_O_5_ glass, a difference is also observed during repeated measurements between the activation energy values of the as-quenched and annealed glasses (0.53 and 0.46 eV, respectively).

A more pronounced and continuous influence on the activation energy values in the high-temperature range, as well as on the electrical conductivity values (σDC at 100 °C in Table 2), is observed in the concentration interval between 0 and 5 mol% ZnO. At concentrations above 5 mol%, the studied glasses already differ from one another in terms of electrical conductivity. When evaluating the measured conductivity values (σDC at 100 °C in Table 2), the usual slight increase during repeated measurements is observed.

### 3.4. Complex Permittivity

Based on measurements of the electrical capacitance C and the loss factor tgδ of glass samples during their stepwise heating, the values of the real and imaginary parts of the complex permittivity were determined. The value of the real part of the complex permittivity ε′ was determined by

(2) $$\varepsilon^{\prime }=\frac{Cd}{A\varepsilon_{0}},$$

where C is the capacitance of the sample, d is the thickness of the sample, A is the area of the cross-section of the sample, and ε_0_ is the permittivity of vacuum.

The value of the imaginary part of the complex permittivity ε″ was determined using the equation

ε″ = ε′ tgδ,(3)

where tgδ is the measured loss factor of the sample.

**Figure 3:**
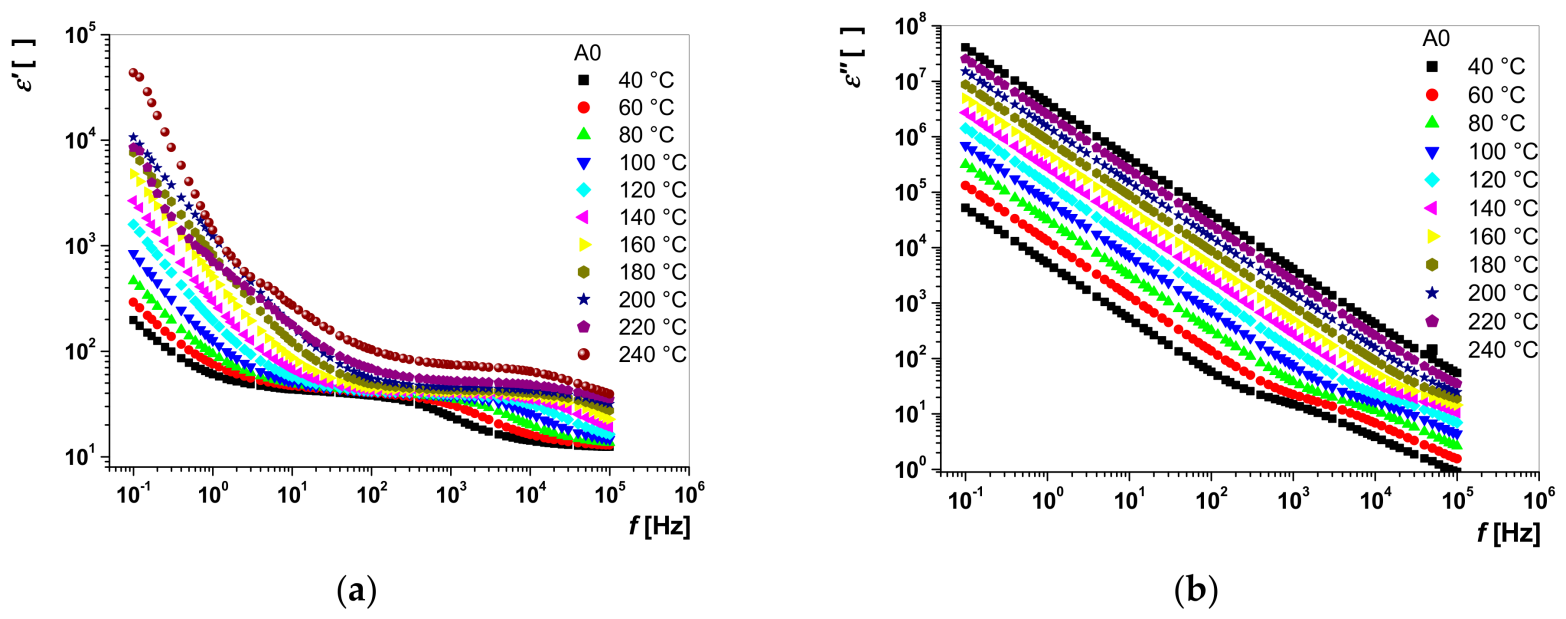
Frequency dependencies of the real (**a**) and imaginary (**b**) parts of the complex permittivity of the glass A0 (35BaO-65V_2_O_5_) measured at various temperatures.

Figure 3 shows the frequency dependences of the real and imaginary parts of the complex permittivity of the 35BaO-65V_2_O_5_ (A0) glass measured at different temperatures. In the observed frequency interval (0.1 Hz to 100 kHz), two significant decreases in the values of the real part of the complex permittivity (Figure 3a) occur. The first decrease at frequencies up to 100 Hz is significantly temperature-dependent, while with increasing temperature, this decrease shifts to higher frequencies. The second decrease in the measured curves of the real part of the complex permittivity is observable at frequencies above 100 Hz and is again temperature-dependent. Both decreases represent two different effects, which are visible in the frequency dependences of the imaginary part of the complex permittivity (Figure 3b).

**Figure 4:**
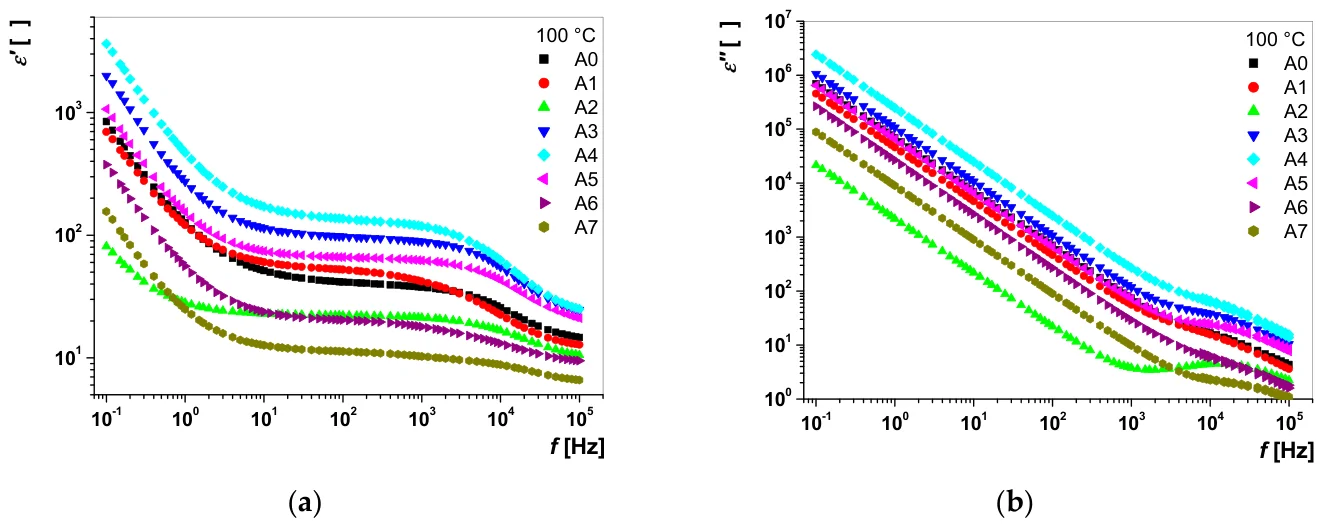
Frequency dependencies of real (**a**) and imaginary (**b**) parts of complex permittivity of the various ZnO–BaO–V_2_O_5_ glasses measured at 100 °C.

**Figure 5:**
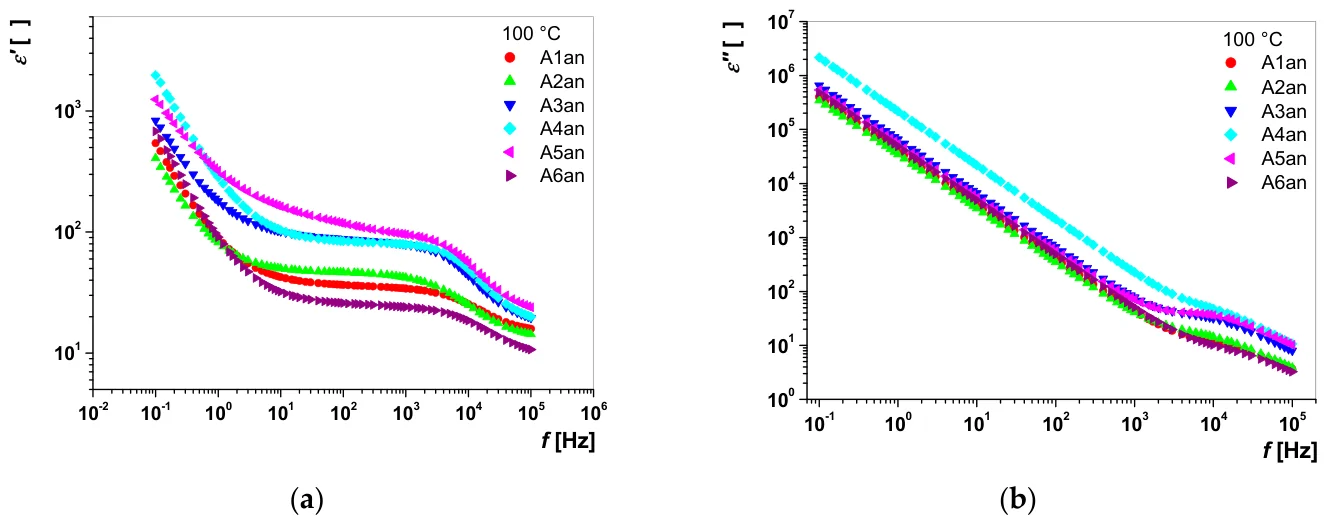
Frequency dependencies of real (**a**) and imaginary (**b**) parts of complex permittivity of the various annealed ZnO–BaO–V_2_O_5_ glasses measured at 100 °C.

The existence of two dielectric relaxation mechanisms in the frequency dependences of the real and imaginary parts of the complex permittivity is also observed in ZnO-containing glasses (Figure 4 and Figure 5 for the annealed glasses). The chemical composition of the glasses (ZnO content) does not have a significant effect on the frequency shift in either mechanism in the frequency dependences of the real and imaginary parts of the complex permittivity.

The effect of annealing during glass preparation can be seen by comparing Figure 4 and Figure 5. Slight differences are observed in the frequency dependences of the real part of the complex permittivity (part (a) of both figures). Significant changes are observed in the frequency dependences of the imaginary part of the complex permittivity (part (b) of both figures). After annealing, changes are observed in the dispersion of the imaginary part of the complex permittivity at lower frequencies, corresponding to the aforementioned first mechanism.

### 3.5. Impedance Spectra

**Figure 6:**
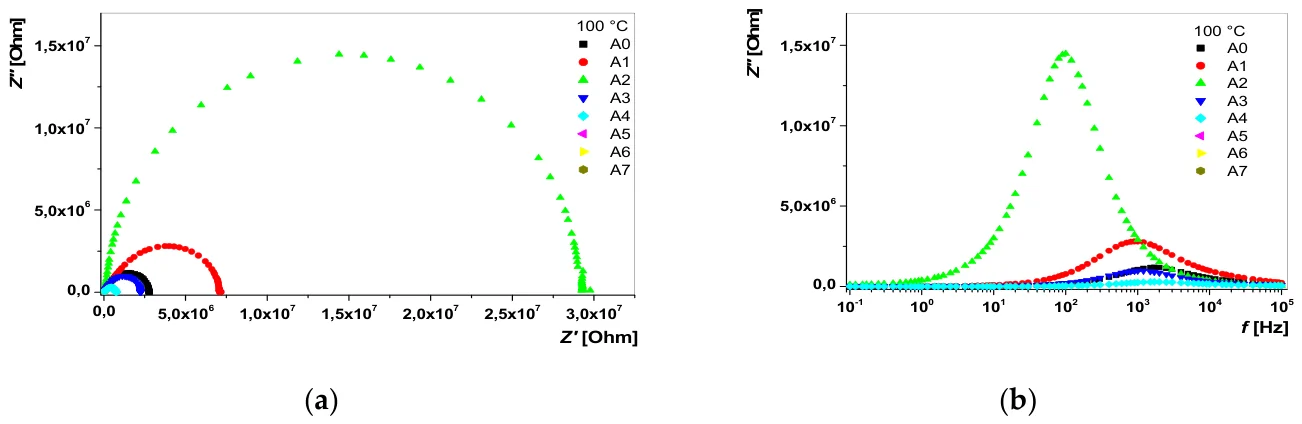
Impedance spectra of the various ZnO–BaO–V_2_O_5_ glasses measured at 100 °C: (**a**) complex plane plots, (**b**) frequency dependencies of the imaginary part of complex impedance.

**Figure 7:**
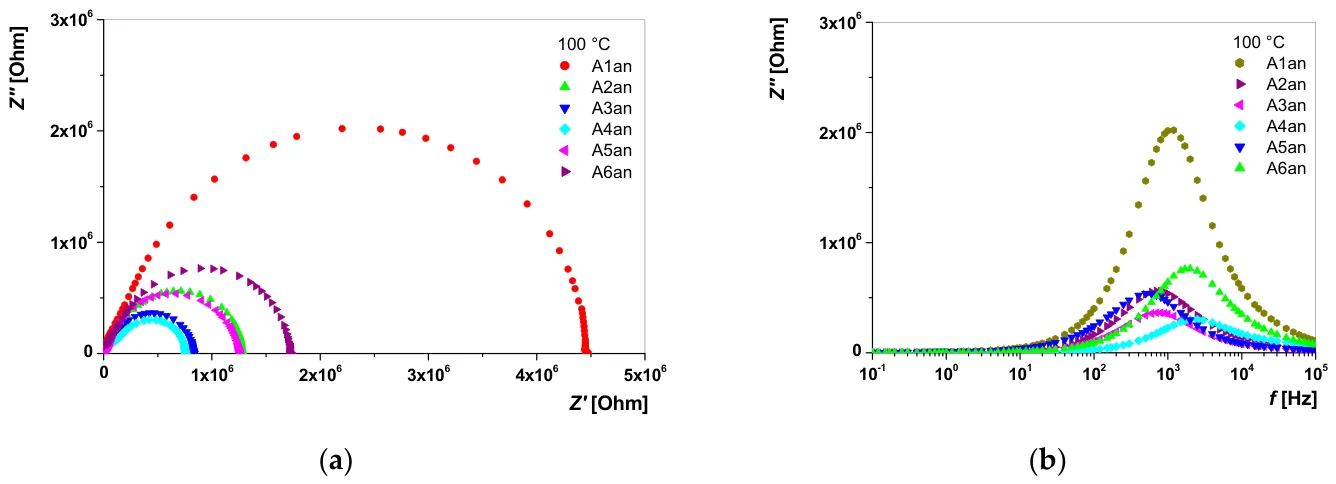
Impedance spectra of the annealed ZnO–BaO–V_2_O_5_ glasses measured at 100 °C: (**a**) complex plane plots, (**b**) frequency dependencies of the imaginary part of complex impedance.

For a more detailed characterization of the dielectric response, impedance spectroscopy analysis of the measured samples can be used. Based on the measured electrical capacitance and resistance of the samples at a given frequency, their impedance values were determined. Figure 6 and Figure 7 show the impedance spectra of the as-quenched (Figure 6) and annealed glasses (Figure 7), presented in the complex plane and as frequency dependences of the imaginary part of the impedance.

The shape of the impedance spectra in the complex-plane plots (part (a) of Figure 6 and Figure 7) suggests that an equivalent circuit characterizing the behavior of the samples in an alternating electric field could include a parallel connection of a capacitor C (or a CPE element) and a resistor R.

**Figure 8:**
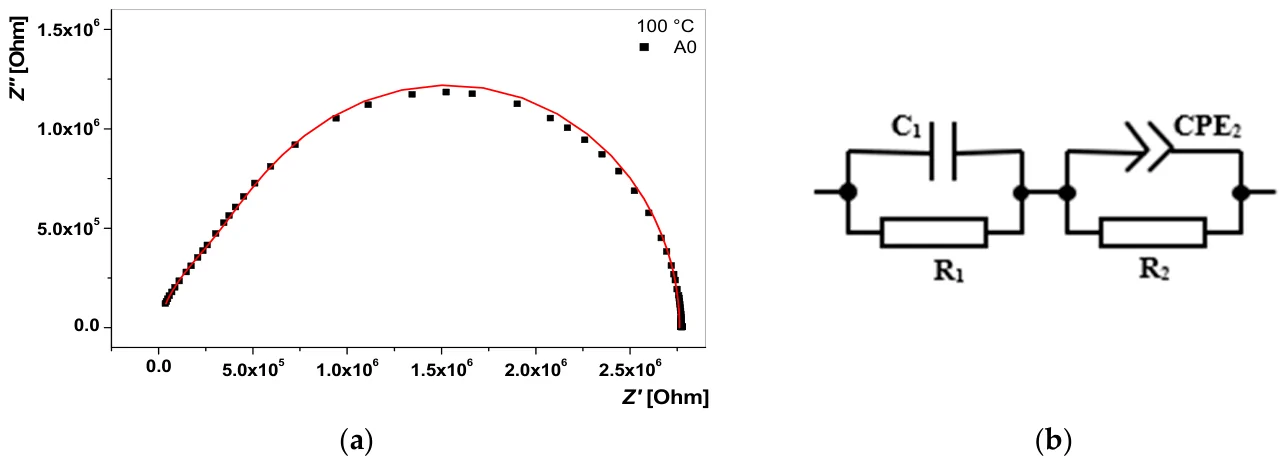
(**a**) Impedance spectra of A0 glass (35BaO-65V_2_O_5_) measured at 100 °C, with the approximation (red line) obtained using the equivalent circuit (**b**).

An equivalent circuit consisting of four elements was proposed based on the previous information obtained from the frequency dependences of the complex permittivity regarding the probable occurrence of multiple dielectric relaxation mechanisms, as well as on the mathematical analysis of the measured impedance dependences using the EIS Spectrum Analyser software 1.0 [16]. Figure 8 presents an example of the approximation results for the 35BaO–65V_2_O_5_ glass (A0), together with a schematic representation of the equivalent circuit used. The equivalent-circuit parameters determined for all glass samples are summarized in Table 3.

A CPE element was also incorporated into the proposed equivalent circuit. The impedance of a CPE element is defined by the following relation [17]:

Z_CPE_(ω) = *P*^−1^ (jω)^−*n*^ (4)

where ω is the angular frequency, *P* and *n* (0≤n≤1) are the parameters whose values are determined by a fitting procedure. For *n* = 1, CPE describes an ideal capacitor of the capacity *C* = *P*, and for *n* = 0, CPE stands for an ideal resistor of the resistivity *R* = 1/*P*.

The values of the deviation parameter *n* lower than one also indicate a certain degree of inhomogeneity in the structure. This means that by comparing the values obtained by approximation, it is possible to compare different glasses in terms of the homogeneity of their structure, or the effect of annealing. Although the values of the parameters of the individual members of the equivalent circuit were determined for all glasses with an uncertainty not exceeding 4%, it is relatively difficult to compare individual glasses. Separation into multiple mechanisms (similarly to the case of the frequency dependences of the complex permittivity) is very difficult for the frequency dependences of the imaginary part of the impedance (Figure 6b and Figure 7b).

### 3.6. Modulus Spectra

From the perspective of separating and possibly quantifying individual dielectric relaxation mechanisms, modulus spectroscopy can be used. This technique analyses the values of the complex electric modulus M*. The complex electric modulus M* is defined as the reciprocal of the complex permittivity ε*. The real ε′ and imaginary ε″ parts of the complex permittivity correspond to the relative dielectric constant and dielectric loss factor, respectively [15]. One advantage of using the complex electric modulus is that the modulus spectrum effectively suppresses information related to high-capacitance electrode effects [18]. When only one dielectric relaxation mechanism characterized by a single relaxation time exists (the “ideal case”), the relaxation peak in the frequency dependence of the imaginary part of the complex electric modulus, M″ = M″(f), has the shape of a Debye distribution, while the complex electric modulus plotted in the complex plane has the shape of a semicircle.

**Figure 9:**
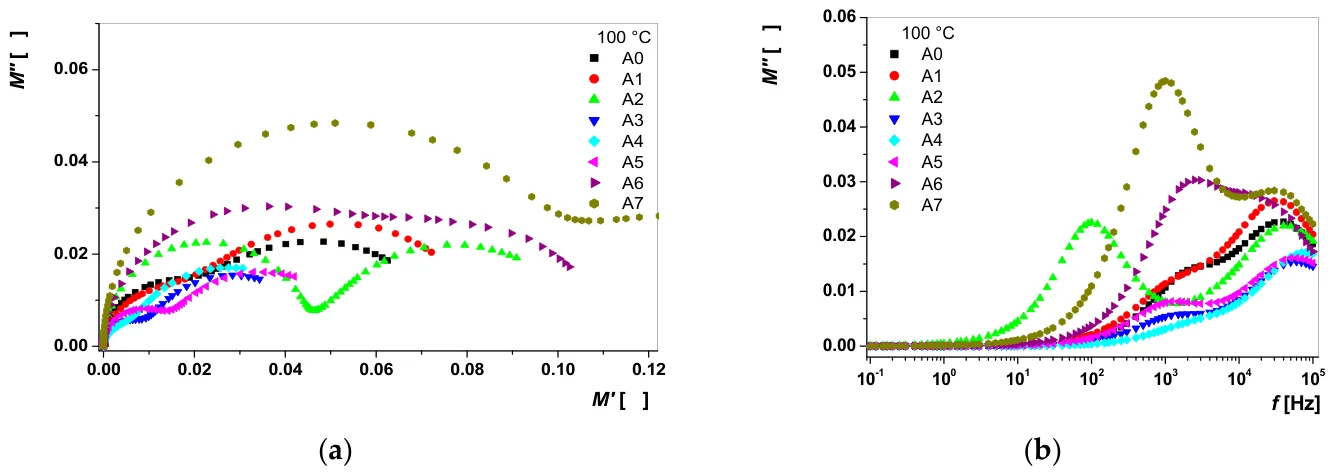
Modulus spectra of the various ZnO–BaO–V_2_O_5_ glasses measured at 100 °C: (**a**) complex plane plots, (**b**) frequency dependencies of the imaginary part of complex electric modulus.

**Figure 10:**
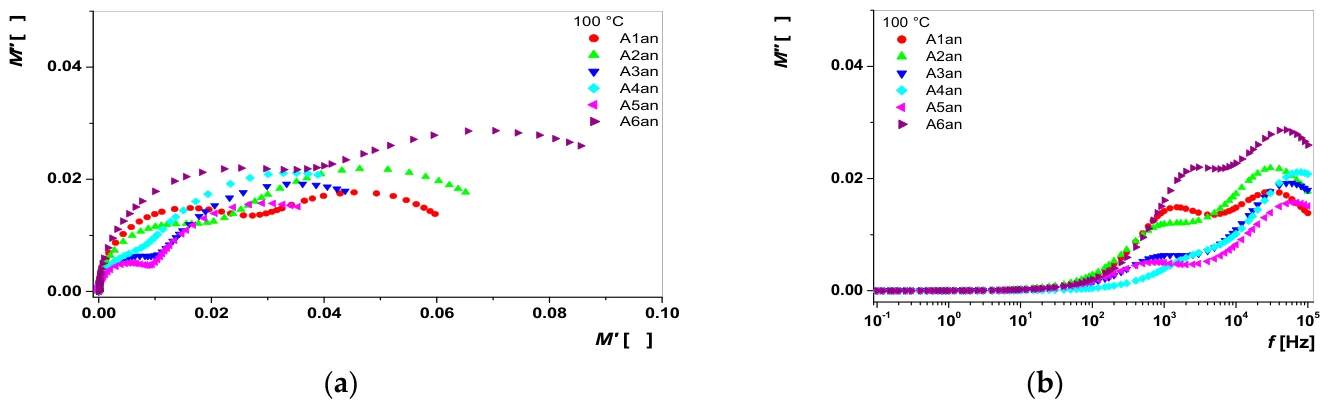
Modulus spectra of the various annealed ZnO–BaO–V_2_O_5_ glasses measured at 100 °C: (**a**) complex plane plots, (**b**) frequency dependencies of the imaginary part of complex electric modulus.

When multiple dielectric relaxation mechanisms exist, the measured complex-modulus curves are deformed both in the complex plane and in their frequency dependences. Figure 9 and Figure 10 show the complex electric modulus dependences of the studied as-quenched and annealed glasses at 100 °C. As can be seen from the complex-plane plots (part (a) of the figures), similarly to the complex permittivity, the measured curves can be divided into separate mechanisms manifested as superimposed parts of semicircles. Similarly, in the frequency dependences of the imaginary part of the complex electric modulus (part (b) of the figures), the dielectric relaxation mechanisms can be clearly distinguished as a superposition of several peaks.

**Figure 11:**
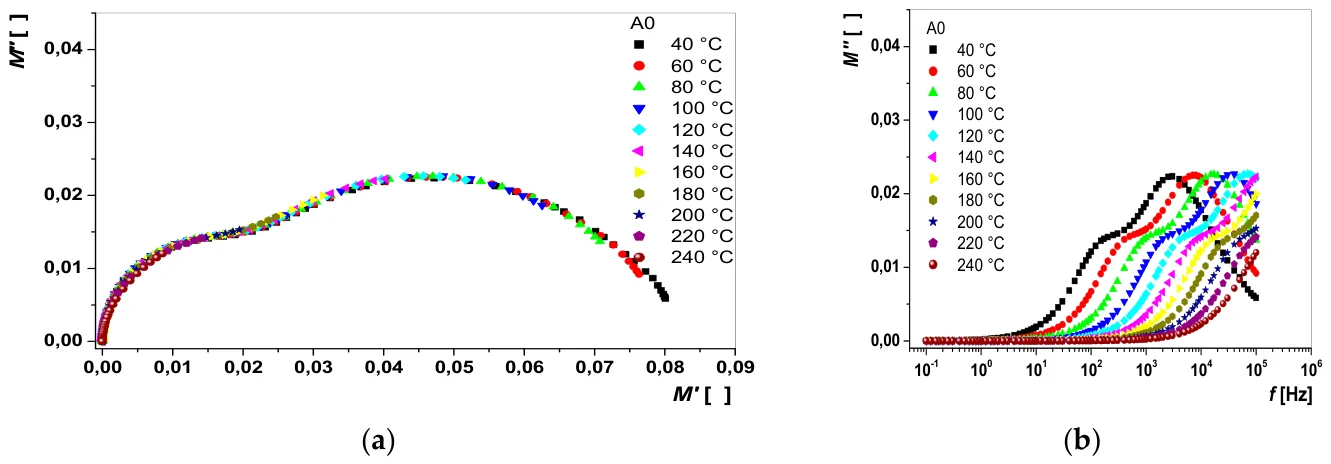
Modulus spectra of A0 glass (35BaO-65V_2_O_5_) measured at various temperatures: (**a**) complex plane plots, (**b**) frequency dependencies of the imaginary part of complex electric modulus.

The advantage of using complex electrical modulus measurements is the possibility of monitoring the temperature shift in multiple mechanisms. In Figure 11, there is an example of displaying the measured values of the complex electrical modulus of the 35BaO-65V_2_O_5_ (A0) glass measured at different temperatures. The shape of the measured dependence of the complex electrical modulus displayed in the complex plane (part (a) of the figure) is preserved during temperature changes, which confirms that the measured glass is thermally stable. The frequency dependences of the imaginary part of the complex electrical modulus (part (b) of the figure) show a mutual frequency shift in the measured dependences at different temperatures. The shape of these frequency dependences is given by the superposition of two maxima. Peaks were fitted by the equation derived from [19]:

(5) $$M^{\prime }=M^{*}\frac{{\left(2\pi f\cdot \tau \right)}^{\beta }\sin\frac{\pi \beta }{2}}{{\left[1+{\left(2\pi f\cdot \tau \right)}^{\beta }\cos\frac{\pi \beta }{2}\right]}^{2}+{\left[{\left(2\pi f\cdot \tau \right)}^{\beta }\sin\frac{\pi \beta }{2}\right]}^{2}}$$

where f is the frequency, τ is the relaxation time value, and β is a constant determining the degree of inhomogeneity. For β = 1, the peak has a shape corresponding to the ideal Debye distribution; for β < 1, the peak can be characterized as a narrow spectrum of close relaxation time values. M* is a constant characterizing the magnitude of the given peak.

**Figure 12:**
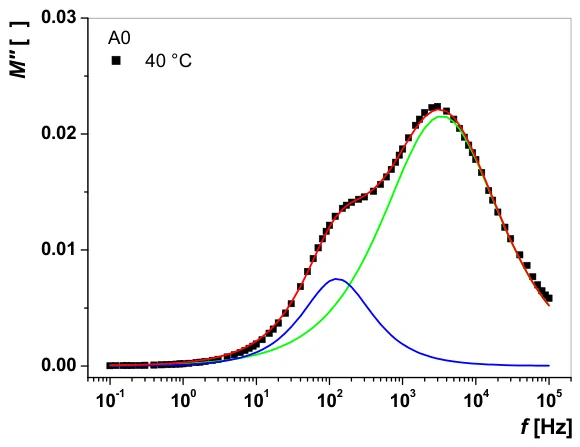
Frequency dependences of the imaginary part of the complex electrical modulus of A0 (35BaO-65V_2_O_5_) glass measured at 40 °C with a display of separation into two maxima—low-frequency peak (solid blue line) and high-frequency peak (solid green line) (solid red line). The sum of the contributions of both peaks is shown by the solid red line.

Figure 12 is an example of the deconvolution of the frequency dependence of the imaginary part of the complex electrical modulus for 35BaO-65V_2_O_5_ (A0) glass measured at 40 °C using Equation (5). The superposition of the assumed peak (red solid line) corresponds well with measured values. Separate peaks could be characterized based on the deconvolution of all measured dependencies M″ (f) of all glasses. The first low-frequency peak is less pronounced and has a Debye shape (β = 1). The second dominant high-frequency peak is slightly expanded, and it is represented by spectra of close relaxation times (β is in the interval from 0.7 to 0.8).

**Figure 13:**
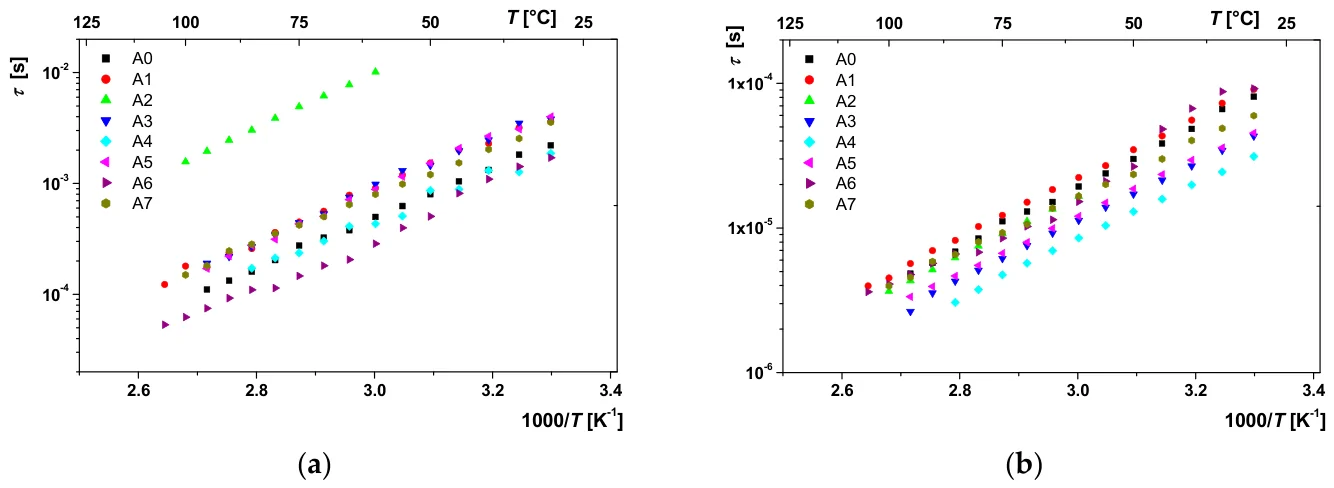

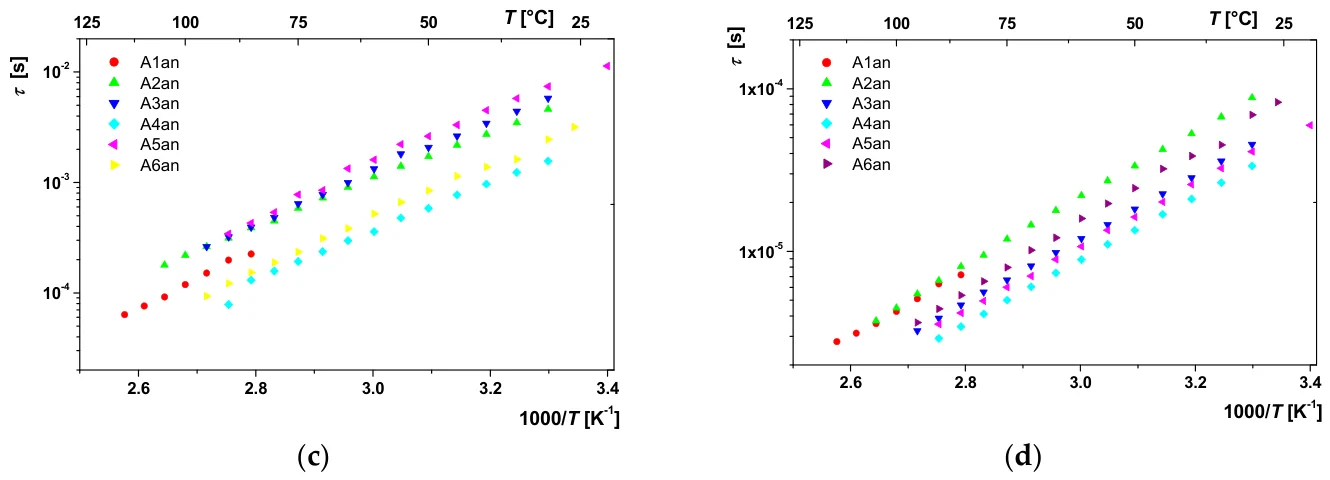
Temperature dependences of relaxation times of the corresponding fitted peaks: (**a**) low-frequency peak of ZnO–BaO–V_2_O_5_ glasses, (**b**) high-frequency peak of ZnO–BaO–V_2_O_5_ glasses, (**c**) low-frequency peak of annealed ZnO–BaO–V_2_O_5_ glasses, (**d**) high-frequency peak of annealed ZnO–BaO–V_2_O_5_ glasses.

The value of relaxation times connected with individual peaks could be determined from Equation (5). Figure 13 presents the relaxation times of as-quenched and annealed glasses for the first low-frequency peak (parts (a) and (c) of the figure) and for the second high-frequency peak (parts (c) and (d) of the figure). Temperature dependences of relaxation times plotted in Figure 13 have an Arrhenius behavior according to

(6) $$\tau =\tau_{0}e^{\frac{E\tau }{kT}}$$

where Eτ is the activation energy of the temperature change in the relaxation time of the relevant peak, τ0 is the pre-exponential factor, k is the Boltzmann constant, and T is the temperature.

From the linear parts of the temperature dependences of the determined relaxation times (Figure 13), the activation energies of the relaxation times of both peaks and the corresponding pre-exponential factors were determined by approximation using relation (6). The results of this approximation are summarized in Table 4.

From the point of view of the mutual comparison of the values in Table 4, it can be seen that the effect of annealing does not have a significant effect on the dielectric relaxation. This fact is manifested by small differences between the respective values of activation energies and pre-exponential factors between the as-quenched and the annealed ZnO–BaO–V_2_O_5_ glasses. Some larger differences in the values of the low-frequency peak are associated with its small magnitude and insignificance in some glasses, which causes an increase in the uncertainty of the determination of the activation energy of the relaxation time and the pre-exponential factor. For this reason, it is also difficult to determine more clearly the influence of the change in chemical composition on the low-frequency, less pronounced peak. In the case of the more dominant high-frequency peak, it is possible to observe an agreement between the values of the activation energy and the pre-exponential factor between the glasses and the annealed ones, allowing also the evaluation of the influence of the chemical composition on this part of the dielectric relaxation.

The activation energy of the relaxation time of the high-frequency peak gradually decreases with increasing ZnO concentration from 0.42 eV up to a concentration of 7 mol% ZnO with an activation energy of 0.38 eV. With a further increase in the ZnO concentration to 15 mol%, a more significant increase in the activation energy occurs during glass annealing.

### 3.7. Raman Spectra

**Figure 14:**
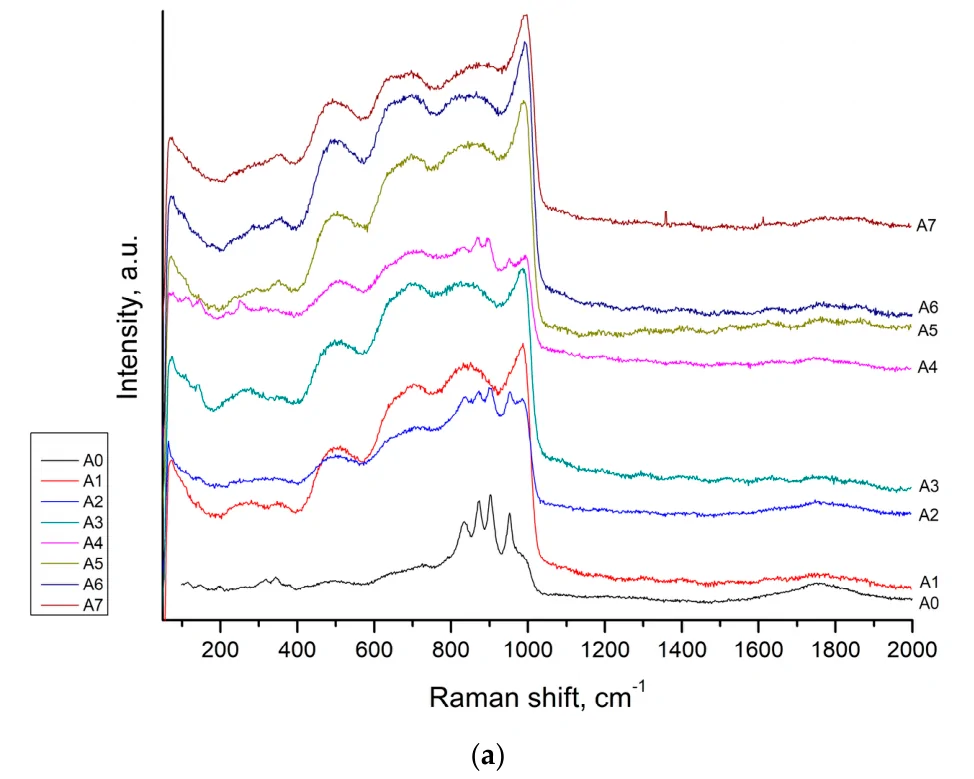

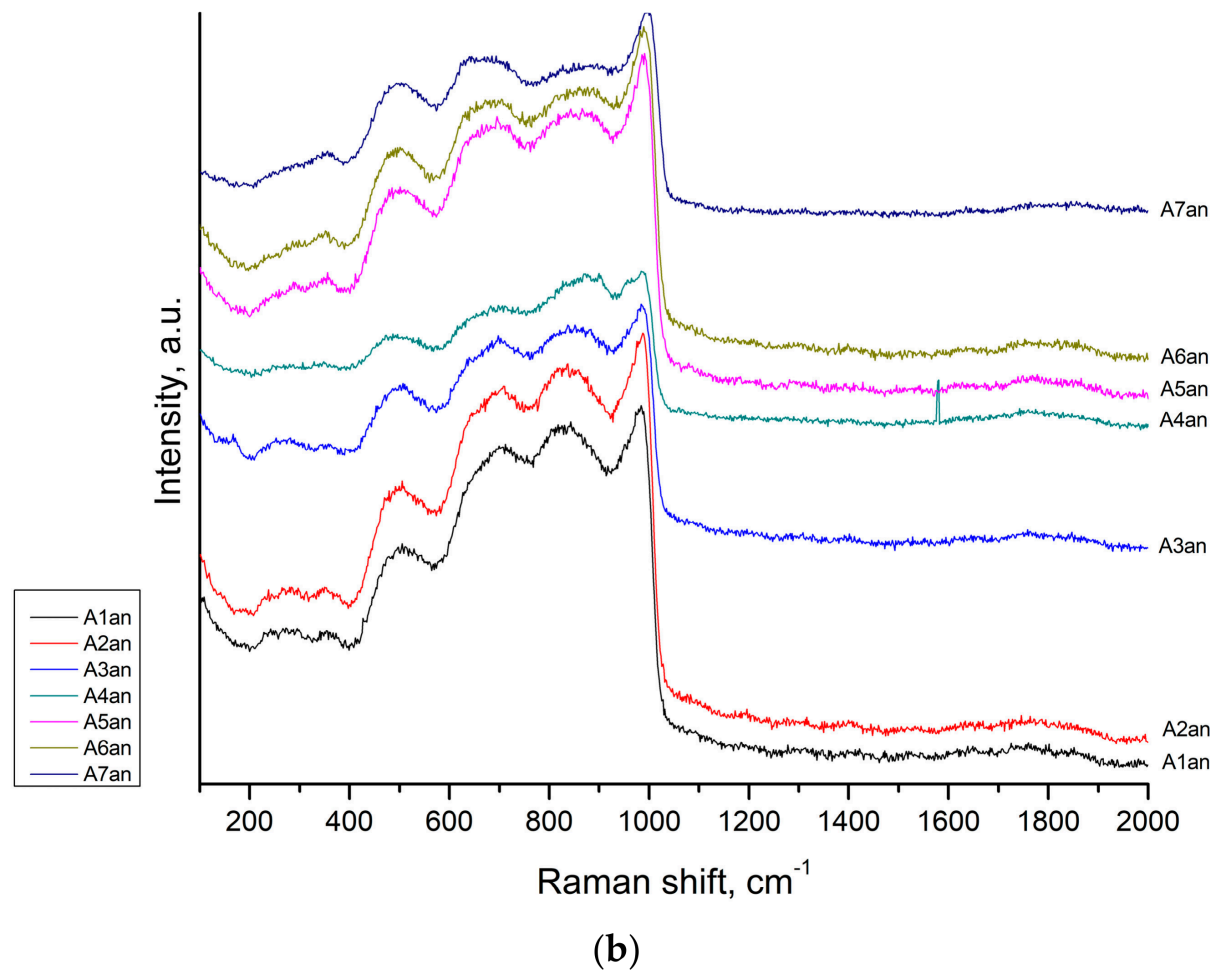
Raman spectra of (**a**) as-quenched and (**b**) annealed glasses in the ZnO–BaO–V_2_O_5_ system.

The Raman spectra of the glasses are presented in Figure 14. Both spectra, as-quenched and annealed, consist of broad and overlapping bands characteristic of an amorphous structure. The main spectral band is a strong, wide band centered at approximately 950–990 cm^−1^; broad bands around 830–870 cm^−1^ and 630–700 cm^−1^ are observed, accompanied by a wide intensity distribution around 500 cm^−1^. At lower wavenumbers, below about 400 cm^−1^, weak and diffuse bands are present. The overall shape of the spectra remains similar for all compositions, with only gradual changes in band intensity and slight shifts in position.

The glass network in vanadate systems is generally formed by a combination of VO_5_ and VO_4_ structural units [20,21,22,23]. Previous structural investigations of V_2_O_5_-containing tellurite glasses have shown that vanadium may occur in VO_5_ trigonal bipyramids forming ${\left({VO}_{4}\right)}_{3n}^{n-}$ chains and ${\left(V_{2}O_{8}\right)}_{n}$ zig-zag chains, together with VO_4_ tetrahedral units [23]. The relative abundance and connectivity of these structural species depend strongly on the overall glass composition. The incorporation of modifier oxides changes their distribution and connectivity. Modifier cations may occupy non-network positions or form bonds with oxygen atoms associated with the vanadium polyhedra, thereby modifying the local structure. These changes are manifested by shifts in the IR and Raman band positions and variations in their relative intensities. With increasing modifier concentration, a gradual conversion of VO_5_ units into VO_4_ tetrahedra may occur, reflecting changes in the connectivity of the vanadate network [20,23].

While the literature reports a characteristic band around 800 cm^−1^, attributed to vibrations of isolated vanadate units (VO_4_) and non-bridging oxygen environments, the corresponding band in the present glasses is shifted toward higher wavenumbers (841–873 cm^−1^). This shift suggests a modification of the local structural environment of vanadium caused by the incorporation of ZnO. The formation of V–O–Zn linkages and the consequent alteration of the glass network may increase the bond strength and reduce the local symmetry, leading to the observed frequency increase.

The band observed in the 968–998 cm^−1^ range is assigned to stretching vibrations involving terminal vanadyl (V=O) bonds. Similar bands at approximately 971–1005 cm^−1^ were associated with non-bridging V=O bonds in VO_5_ units in other vanadate glass systems [21,22,23].

Furthermore, the broadening of the bands in the 450–550 cm^−1^ region and the enhanced intensity in the 840–900 cm^−1^ range indicate an increase in structural disorder and the coexistence of different vanadium-oxygen environments. These observations suggest that ZnO acts as a network modifier, promoting depolymerization of the vanadate network and facilitating the formation of mixed V–O–Zn structural units. Overall, the Raman results confirm that the addition of ZnO significantly influences the short-range structure of the glass while preserving the characteristic vanadyl (V=O) groups.

The Raman spectra of the annealed samples exhibit the same main vibrational features as the as-prepared glasses; however, significant changes in band shape, intensity, and resolution are observed after heat treatment. The spectra are characterized by bands located at approximately 260–355, 490–500, 640–700, 830–860, and 980–997 cm^−1^, corresponding to vibrations of V–O, V–O–V, and terminal V=O structural units. Compared with the as-prepared samples, the annealed glasses display noticeably sharper and better-resolved Raman bands. This behavior indicates a reduction in structural disorder and relaxation of frozen-in stresses generated during the rapid quenching process. The enhanced definition of the bands suggests an increase in short-range structural ordering within the glass network. Particularly significant is the development of the Raman band in the 640–700 cm^−1^ region, assigned to stretching vibrations of bridging V–O–V bonds. The increased intensity and improved resolution of this feature indicate a more ordered arrangement of vanadate polyhedra after annealing. Likewise, the band located in the 830–860 cm^−1^ region becomes more pronounced, suggesting a better-defined distribution of VO_4_ units and/or V–O–Zn linkages. The strongest Raman feature remains the band centered near 990 cm^−1^, attributed to the stretching vibration of terminal vanadyl (V=O) bonds. Its position changes only slightly after annealing, indicating that the fundamental vanadyl structural units remain stable during the heat-treatment process. Nevertheless, the narrowing of this band points to a more homogeneous local environment around the vanadium ions. The low-frequency bands between 260 and 355 cm^−1^ also become more clearly resolved following annealing. These bands are generally associated with bending vibrations involving V–O bonds and collective motions of vanadate structural units. Their increased visibility further supports the conclusion that annealing promotes structural relaxation and improved short-range order. Overall, the Raman results demonstrate that annealing does not fundamentally alter the structural units present in the glass network but leads to a more ordered arrangement of these units. The observed spectral changes are consistent with structural relaxation and partial reorganization of the vanadate network, resulting in reduced disorder and enhanced local structural homogeneity.

The Raman spectrum of sample A4 (7 mol% ZnO) exhibits characteristic bands at approximately 355, 492, 692, 830, and 997 cm^−1^, which are attributed to O–V–O bending vibrations, V–O–V bending and stretching modes, distorted VO_4_ structural units, and terminal vanadyl (V=O) bonds, respectively. The simultaneous presence of well-developed bands associated with both bridging V–O–V linkages and non-bridging vanadate units indicates an intermediate structural state of the glass network. Among all investigated compositions, the A4 glass (7 mol% ZnO) appears to represent a structural threshold. In this composition, ZnO significantly modified the vanadate network through the formation of V–O–Zn linkages, while the connectivity of the original V–O–V network remained largely preserved. Consequently, the glass probably contains a balanced distribution of polymerized and depolymerized vanadate structural units. The distinct behavior observed near 7 mol% ZnO is also consistent with our previous infrared-spectroscopic study of the same glass system, where changes in the vanadate vibrational bands were detected from this composition range onward. In particular, the spectral modifications were attributed to changes in the relative populations of VO_4_ and VO_5_ units and to the formation of V–O–Zn linkages. These earlier results support the present interpretation of 7 mol% ZnO as a possible transition region in the short-range structural organization of the glass network rather than as a sharply defined critical composition [13].

These structural features are consistent with the electrical measurements. In the high-temperature region, the activation energy of DC conductivity for the glass containing 7 mol% ZnO changes only slightly between the first and repeated heating cycles, in contrast to the more pronounced changes observed for the other compositions. Impedance analysis also shows a relatively low resistance and a marked increase in the CPE exponent after annealing, indicating a more homogeneous electrical response. Taken together, these results suggest that the composition containing 7 mol% ZnO lies within a transition region in which changes in the glass network begin to affect the electrical transport and dielectric relaxation processes.

The Raman and electrical results therefore indicate that 7 mol% ZnO represents a possible transition region where the vanadate network undergoes substantial structural reorganization. The coexistence of V–O–V and V–O–Zn structural units likely stabilizes the distribution of mixed-valence V^4+^/V^5+^ centers responsible for electron hopping conductivity, giving rise to the distinctive electrical behavior observed for sample A4.

## 4. Conclusions

In this work, DC conductivity measurements, dielectric, impedance and modulus spectroscopy, Raman spectroscopy, X-ray diffraction (XRD), and differential scanning calorimetry (DSC) were used to investigate the effects of ZnO content and thermal treatment on the properties of vanadate glasses. Two series of bulk glasses with compositions xZnO–(35 − x)BaO–65V_2_O_5_ (x = 0–20 mol%) were prepared by the melt-quenching technique: an as-quenched series and an annealed series. XRD confirmed the amorphous structure of both the as-quenched and annealed samples, while DSC analysis of the as-quenched glasses showed that the electrical measurements were performed below the onset of crystallization. Correlations were established between the Raman spectroscopic and electrical results. The combined findings indicate that the composition containing 7 mol% ZnO represents a compositional threshold associated with changes in the electrical and dielectric behavior of the investigated glass system.

The DC conductivity of the glasses in the investigated temperature range of 20–250 °C exhibits two Arrhenius regions: below 120 °C and above 150 °C. The activation energies in the high-temperature region are generally higher than those in the low-temperature region. During the first heating cycle of the as-quenched glasses, activation energies are approximately 0.65–0.82 eV in the high-temperature region, compared with 0.46–0.52 eV in the low-temperature region. In general, repeated heating and annealing decrease the activation energy in the high-temperature region, with values of approximately 0.50–0.68 eV.

AC measurements, analyzed using the complex permittivity, impedance, and electric modulus formalisms, reveal two distinguishable temperature-dependent processes within the frequency range of 0.1 Hz–100 kHz. Both mechanisms are visible in the frequency dependences, especially of the real part of the complex permittivity investigated via modulus spectroscopy. Both processes are particularly evident in the frequency dependence of the real part of the electric modulus. Therefore, the electric modulus formalism enables a clearer quantitative characterization of these processes than impedance analysis. Annealing has a particularly pronounced effect on the frequency dependence of the imaginary part of the complex permittivity at low frequencies.

Two dielectric relaxation processes are distinguished within the investigated frequency range. The less pronounced low-frequency process is approximately described by a single relaxation time and exhibits Arrhenius behavior, with activation energies of 0.43–0.53 eV. The dominant high-frequency process is characterized by a relatively narrow distribution of relaxation times and activation energies of 0.38–0.42 eV. The activation energy of the low-frequency process is comparable to that of DC conductivity in the low-temperature region. However, no clear one-to-one correspondence between the dielectric relaxation processes and DC conductivity was established.

Based on the compositional dependences of the electrical and dielectric properties, three concentration regions can be considered: (1) ZnO (0–5 mol%): The electrical properties change relatively continuously with increasing ZnO content. Repeated heating and annealing generally reduce the activation energy in the high-temperature region. (2) ZnO (7 mol%): This composition shows distinctive behavior compared with the general trends observed for the other glasses. In particular, the high-temperature activation energy changes only slightly between the first and repeated heating cycles, from 0.680 to 0.674 eV for the as-quenched glass. The sample also exhibits the lowest $R_{1}$ value among the as-quenched glasses and an increase in the CPE exponent from 0.782 to 0.851 after annealing. These results indicate a relatively low resistance and a more homogeneous electrical response after heat treatment. ZnO (10–20 mol%): More pronounced variations in the electrical and dielectric parameters are observed in this concentration range. These variations can be associated with the greater replacement of BaO by ZnO and the resulting changes in the local structure of the vanadate network.

The Raman spectra show gradual changes in band position and intensity with increasing ZnO content. The glass network is mainly composed of interconnected VO_5_ and VO_4_ structural units, with terminal V=O bonds remaining characteristic of all investigated compositions. The gradual replacement of BaO by ZnO modifies the connectivity of the vanadate network, probably through changes in the relative distribution of VO_5_ and VO_4_ units and the formation of V–O–Zn linkages. Annealing promotes structural relaxation and improved short-range ordering without fundamentally changing the main structural units.

## Figures and Tables

**Table 1:** Glass compositions, glass-transition temperature Tg, crystallization temperature Tx, and thermal stability ΔT = (Tx_1_ − Tg). All temperatures are reported in °C.

ID	Glass Compositions	Tg, °C	Tx_1_, °C	Tx_2_, °C	Tx_3_, °C	ΔT, °C (Tx_1_ − Tg)
A1	1ZnO-34BaO-65V_2_O_5_	303	358	-	-	55
A2	3ZnO-32BaO-65V_2_O_5_	301	364	-	-	63
A3	5ZnO-30BaO-65V_2_O_5_	299	372	-	-	73
A4	10ZnO-25BaO-65V_2_O_5_	271	321	415	-	50
A5	15ZnO-20BaO-65V_2_O_5_	268	318	388	441	50
A6	20ZnO-15BaO-65V_2_O_5_	262	315	345	-	53

**Table 2:** Conduction activation energies (Eσ), pre-exponential factors (σo), and electrical conductivity (σdc) of ZnO–BaO–V_2_O_5_ glasses at 100 °C in the first and second heating cycles. Index 1 corresponds to temperatures below 120 °C, and index 2 corresponds to temperatures above 150 °C. The accuracy of activation energy is ±0.02 eV for Eσ1 and ±0.03 eV for Eσ2.

	ID	Glass System	Eσ1 [eV]	σ01 [S/m]	Eσ2 [eV]	σ02 [S/m]	σDC (100 °C) [nS/m]
The first cycle	A0	35BaO-65V_2_O_5_	0.51	3.46	0.68	261.1	3840
	A1	1ZnO-34BaO-65V_2_O_5_	0.48	4.739	0.66	449	1734
	A2	3ZnO-32BaO-65V_2_O_5_	0.47	0.497	0.82	5422	1921
	A3	5ZnO-30BaO-65V_2_O_5_	0.46	5.778	0.78	20,377	3138
	A4	7ZnO-28BaO-65V_2_O_5_	0.52	27.3	0.68	2081	2540
	A5	10ZnO-25BaO-65V_2_O_5_	0.5	17.36	0.68	2999	3289
	A6	15ZnO-20BaO-65V_2_O_5_	0.52	17.58	0.655	654.5	1561
	A7	20ZnO-15BaO-65V_2_O_5_	0.43	0.263	0.763	4361	425
The second cycle	A0	35BaO-65V_2_O_5_	0.51	63	0.52	81.6	7461
	A1	1ZnO-34BaO-65V_2_O_5_	0.49	14.74	0.5	17.73	3038
	A2	3ZnO-32BaO-65V_2_O_5_	0.54	8.062	0.6	30.46	431
	A3	5ZnO-30BaO-65V_2_O_5_	0.49	18.73	0.61	430.1	3900
	A4	7ZnO-28BaO-65V_2_O_5_	0.56	208	0.674	2005	3242
	A5	10ZnO-25BaO-65V_2_O_5_	0.53	78.94	0.536	104.3	5243
	A6	15ZnO-20BaO-65V_2_O_5_	0.497	19.5	0.5	21.79	3631
	A7	20ZnO-15BaO-65V_2_O_5_	0.463	1.92	0.56	18.971	1064
The first cycle annealed glasses	A1an	1ZnO-34BaO-65V_2_O_5_	0.46	4.739	0.548	15.52	891
	A2an	3ZnO-32BaO-65V_2_O_5_	0.53	6.15	0.615	48.7	403
	A3an	5ZnO-30BaO-65V_2_O_5_	0.51	11.95	0.606	172.7	1627
	A4an	7ZnO-28BaO-65V_2_O_5_	0.52	27.3	0.548	57.27	2734
	A5an	10ZnO-25BaO-65V_2_O_5_	0.501	3.621	0.653	234.2	611
	A6an	15ZnO-20BaO-65V_2_O_5_	0.461	3.722	0.603	182.5	2178
The second cycle annealed glasses	A1an	1ZnO-34BaO-65V_2_O_5_	0.485	4.185	0.54	18.46	1142
	A2an	3ZnO-32BaO-65V_2_O_5_	0.546	21.33	0.546	18.21	865
	A3an	5ZnO-30BaO-65V_2_O_5_	0.52	59.871	0.536	89.939	5696
	A4an	7ZnO-28BaO-65V_2_O_5_	0.56	213.9	0.53	55.177	4237
	A5an	10ZnO-25BaO-65V_2_O_5_	0.46	3.028	0.573	63.841	2028
	A6an	15ZnO-20BaO-65V_2_O_5_	0.473	12.1	0.49	20.263	4746

**Table 3:** Approximated parameters of impedance spectra at 100 °C for investigated glasses using the equivalent circuit in Figure 8b. The accuracy of calculated parameters is within 5%.

Glass	*R*_1_	*C*_1_	*R*_2_	*P*_2_	*n*_2_
A0	2.341 × 10^6^	4.1567 × 10^−11^	4.2159 × 10^5^	1.0982 × 10^−10^	0.85807
A1	4.1637 × 10^6^	4.813 × 10^−11^	2.9525 × 10^6^	3.789 × 10^−10^	0.70164
A2	2.8915 × 10^7^	5.4455 × 10^−11^	1.8028 × 10^5^	5.9613 × 10^−10^	0.81306
A3	1.9856 × 10^6^	7.3214 × 10^−11^	2.4567 × 10^5^	2.1987 × 10^−10^	0.82182
A4	5.6311 × 10^5^	1.63 × 10^−10^	1.8303 × 10^5^	4.5589 × 10^−10^	0.78197
A5	2.3821 × 10^6^	7.0537 × 10^−11^	4.7076 × 10^5^	3.3418 × 10^−9^	0.63043
A6	5.5609 × 10^6^	2.4777 × 10^−11^	4.2811 × 10^6^	7.3373 × 10^−11^	0.83193
A7	7.5717 × 10^6^	2.6907 × 10^−11^	3.2419 × 10^6^	1.6733 × 10^−10^	0.84475
A1an	3.7227 × 10^6^	4.0501 × 10^−11^	7.3726 × 10^5^	9.4504 × 10^−10^	0.71369
A2an	1.0087 × 10^6^	2.3045 × 10^−10^	2.7685 × 10^5^	4.0563 × 10^−9^	0.68838
A3an	2.8915 × 10^7^	5.4455 × 10^−11^	1.8027 × 10^5^	5.9594 × 10^−10^	0.81309
A4an	5.9183 × 10^5^	1.175 × 10^−10^	1.6073 × 10^5^	1.6371 × 10^−10^	0.85141
A5an	9.1988 × 10^5^	3.4021 × 10^−10^	3.5334 × 10^5^	2.398 × 10^−8^	0.57179
A6an	1.3452 × 10^6^	6.566 × 10^−11^	3.7857 × 10^5^	1.3619 × 10^−9^	0.71303

**Table 4:** Activation energies of the temperature change in the relaxation time for the low-frequency (Eτ1) and high-frequency (Eτ2) peaks for dependencies M″(f), pre-exponential factors (τ01 and τ02) of ZnO–BaO–V_2_O_5_ glasses. The accuracy of activation energies is ±0.02 eV for Eτ2 and ±0.03 eV for Eτ1.

	ID	Glass System	*E*_τ1_ [eV]	τ_01_ [s]	*E*_τ2_ [eV]	τ_02_ [s]
	A0	35BaO-65V_2_O_5_	0.45	3.63 × 10^−11^	0.42	8.71 × 10^−12^
	A1	1ZnO-34BaO-65V_2_O_5_	0.49	3.63 × 10^−11^	0.41	1.23 × 10^−11^
	A2	3ZnO-32BaO-65V_2_O_5_	0.50	3.09 × 10^−10^	0.41	1.17 × 10^−11^
	A3	5ZnO-30BaO-65V_2_O_5_	0.48	4.90 × 10^−11^	0.39	1.20 × 10^−11^
	A4	7ZnO-28BaO-65V_2_O_5_	0.43	1.66 × 10^−10^	0.38	1.48 × 10^−11^
	A5	10ZnO-25BaO-65V_2_O_5_	0.50	2.69 × 10^−11^	0.39	1.28 × 10^−11^
	A6	15ZnO-20BaO-65V_2_O_5_	0.47	2.54 × 10^−11^	0.38	2.63 × 10^−11^
	A7	20ZnO-15BaO-65V_2_O_5_	0.43	2.75 × 10^−10^	0.38	3.16 × 10^−11^
Annealed glasses	A1an	1ZnO-34BaO-65V_2_O_5_	0.53	7.94 × 10^−12^	0.42	9.33 × 10^−12^
	A2an	3ZnO-32BaO-65V_2_O_5_	0.43	3.89 × 10^−10^	0.42	1.32 × 10^−11^
	A3an	5ZnO-30BaO-65V_2_O_5_	0.46	1.35 × 10^−10^	0.39	1.70 × 10^−11^
	A4an	7ZnO-28BaO-65V_2_O_5_	0.43	1.07 × 10^−10^	0.38	1.41 × 10^−11^
	A5an	10ZnO-25BaO-65V_2_O_5_	0.49	5.75 × 10^−11^	0.39	1.32 × 10^−11^
	A6an	15ZnO-20BaO-65V_2_O_5_	0.47	3.80 × 10^−11^	0.42	6.46 × 10^−12^

## Data Availability

The original contributions presented in this study are included in the article. Further inquiries can be directed to the corresponding author.
